# Changes in mixed ethnicity households and neighbourhood transitions in England and Wales

**DOI:** 10.1002/psp.2745

**Published:** 2023-12-14

**Authors:** Gemma Catney, Mark Ellis, Richard Wright

**Affiliations:** 1 Geography, School of Natural and Built Environment, Queen’s University Belfast, Belfast, UK; 2 Department of Geography and Center for Studies in Demography and Ecology, University of Washington, Seattle, Washington, USA; 3 Department of Geography, Dartmouth College, Hanover, New Hampshire, USA

**Keywords:** diversity, ethnicity, households, mixing, neighbourhoods, race

## Abstract

Conventional explanations of neighbourhood ethnic transitions consider what drives differential growth in ethnic group populations without regard to household composition. We enrich these nonhousehold approaches by using consistent Census data on neighbourhoods and households for England and Wales for 2001, 2011 and 2021 to analyse connections between mixed‐ethnicity households and neighbourhood ethnic diversity. We employ a neighbourhood typology of ethnic diversity that identifies neighbourhoods as either low‐ or moderate‐diversity, or high‐diversity, where no single ethnic group is in the majority. We focus particularly on White‐majority and highly diverse neighbourhoods given the dominance of the former in residential spaces in England and Wales, and because they are the principal source of transitions to highly diverse neighbourhoods. Mixed‐ethnicity households have become an increasingly important feature of the ethnic diversification of England and Wales; by 2021, almost 15% of multiperson households were mixed, a growth from 12% in 2011 and 9% in 2001. We find that higher levels of neighbourhood ethnic diversity are associated with higher shares of mixed‐ethnicity households. In high‐diversity neighbourhoods, for example, around 30% of multiperson households (nearly a quarter of a million households) were ethnically mixed in 2021. Levels of household mixing in moderate‐diversity White neighbourhoods were considerably higher than in low‐diversity White neighbourhoods. The same is found for diversifying neighbourhoods. Neighbourhoods that become more ethnically diverse were typically home to higher rates of mixed‐ethnicity households. Stably moderately diverse White neighbourhoods also had a higher proportion of mixed households. Studies of neighbourhood transition that focus on individuals in neighbourhoods may be missing important processes operating in the intimate spaces of the home. Including this intermediate scale of analysis adds to our understanding of neighbourhood ethnic mixing and processes of integration.

## 1 | INTRODUCTION

If few studies of residential racial and ethnic neighbourhood segregation directly address the issue of spatial scale (Johnston et al., 2018), it is probably fair to say that even fewer studies have wrestled with scale and racial and ethnic neighbourhood *diversity*. The analysis in this paper centres on such diversity in England and Wales at two specific levels: neighbourhoods and households. In many countries in the global north, neighbourhoods are becoming more ethnically mixed, and the United Kingdom is no exception (Catney, 2016a; Catney et al., 2023; Johnston et al., 2013; Smith & Simpson, 2015). At the same time, the share of ethnically mixed households is also on the rise (ONS, 2014, 2022). We are interested in the interplay between these two, and specifically the place of mixed‐ethnicity households in neighbourhood racial and ethnic transitions.

Neighbourhood change research has tended to focus on individuals, both empirically (e.g., counts of people in a place) and theoretically (e.g., personal preferences). If households are mentioned, they often appear like individuals in these sorts of studies, as mono‐racial entities such as White, Asian, Black and so on (Gabriel & Spring, 2019). In this study, we think about households as not only mono‐racial but also taking other forms. Such structures include mixed‐ethnicity parentage, ethnically mixed couples, housemates with different ethnicities (i.e., ‘mixed intergenerational groupings’), as well as other household compositions. Such collectivities are not a trivial household form: together, mixed‐ethnicity households (here-after mixed households) comprised 15% of all multiperson house-olds in England and Wales in 2021.^1^ Our analysis of the types of neighbourhoods in which we find such households presents both the chance to better understand the basic geographies of mixed households, as well as how *layers* of mixing—bodies in households, in neighbourhoods—coexist and interrelate. These issues coalesce in our central research question: Is the new neighbourhood diversity of many cities in England and Wales associated with mixing in intimate spaces?

By exploring the conjunction of household and neighbourhood processes of ethnic mixing, we also aim to amplify understandings of neighbourhood *transition* processes. This standpoint allows us to respond to debates that frame demographic and ethnic/racial neighbourhood change as some sort of potential threat to White British identity and sustainability. UK policy‐political‐public narratives often draw attention to neighbourhood change because of the in‐migration by racially minoritised people and families (which are presumed to be mono‐racial). Our approach shifts perspective to account for the fact that neighbourhood change also stems from mixing *within households*. Given that two‐fifths of all people in interethnic relationships in 2011 included someone identifying as White British (ONS, 2014), love in intimate spaces between the majority and minoritised groups likely plays a role in the ethnic and racial changes in (White British) majority‐dominated neighbourhoods. This idea of neighbourhood transition generated by *White British* mixing with racialized ‘others’ within the home requires a reassessment of conventional theory, the most widespread of which is neighbourhood ‘invasion’ as force in neighbourhood change.

A shift in focus from individuals to households is thus not just a shift in scale of analysis (Tindale et al., 2014, p. 393). It brings to the fore an important intervention in the demography of neighbourhood integration. Even residents of diverse neighbourhoods can experience minimal or no interethnic mixing if their household arrangements are mono‐racial (Bonilla‐Silva, 2021). In contrast, household arrangements in a neighbourhood that involve ethnic and racialised groups sharing the intimate space of the home presents a fundamentally alternative understanding of living with difference.

We begin with a review of the largely separate literatures on ethnic and racial mixing in neighbourhoods and households. We then discuss how mixing within households and transitions at the neighbourhood scale may intersect. These ideas and findings motivate our empirical assessment of how much mixing in households affects neighbourhood ethnic transitions, especially the role such intimate mixing plays in the growth of diversity in White‐dominated residential spaces. Our conclusions call (again) for an increased role of the household, and especially household‐scale ethnic mixing, in urban processes, and outlines potential research directions.

## 2 | ETHNIC AND RACIAL MIXING AND NEIGHBOURHOOD TRANSITIONS

Explanations of the process of neighbourhood ethnic transition often trace back to the old, yet still influential, Chicago School human ecology model of invasion‐succession, which describes how the influx of a new group into a neighbourhood stimulates the exit of the existing group (Park, 1952; Park et al., 1925). In this framing, mixing or the presence of multiple groups in a neighbourhood is a temporary state, as the residential space switches from one group to another. Chicago School scholars applied this thinking to the residential spaces of European immigrant groups and Black internal migrants in early 20th‐century Chicago by assuming, wrongly, that the processes mitigating against neighbourhood mixing of the former were a less vigorous version of those affecting the latter (Philpott, 1978). Over subsequent decades, as the distinctions among European immigrant groups faded, the consequences of this failure to set apart the racial exclusion of Black people from the assimilative processes operating on European ethnics became clearer. After mid‐century, invasion‐succession framings of neighbourhood transition focused on how certain White neighbourhoods, filled with mixtures of the descendants of European ethnics, became Black‐majority spaces, often in short order.

The copresence of White and Black people was theorised as temporary because many Whites hold racist fears of the consequences of residential mixing with Black people in terms of prejudiced stereotypes around crime, violence, and home upkeep. In addition, many White people saw intermixing in neighbourhoods as one step removed from more intimate intermixing—a threat not only to neighbourhoods but also the White race (Ellen, 2000, p. 39). Later variants of these ideas included tipping point theory (Schelling, 1971) and White flight more generally (Frey, 1979). The literature has evolved to accommodate neighbourhood formations adapted to the growth of Asian, Latinx and Black groups relative to the White population (e.g., Holloway et al., 2012; Logan & Zhang, 2010). Explanations for observed neighbourhood transitions, nevertheless, still gravitate towards White *intolerance* for the presence of racial and ethnic others in their residential environs, especially Black people (Wright & Ellis, 2021).

The Anglophone scholarly literature on ethnic neighbourhood transition also continues to have a US bias, such that Lupton and Power (2004, p. 7) commented on the paucity of broad‐scale investigations on changing ethnic composition and neighbourhoods in comparison to the United States. UK research on neighbourhood transitions has a stronger focus on social class, especially on gentrification and socioeconomic disadvantage (e.g., Bailey & Minton, 2018; McLachlan & Norman, 2021), and, more recently, on trajectories of neighbourhood deprivation (Lloyd et al., 2023; Patias et al., 2022), and household overcrowding (Lloyd & Gleeson, 2022), than on ethnicity or race. Empirically, scholars also tend toward case studies of particular neighbourhoods rather than broad‐scale research (Tunstall, 2016). Academic inquiries have focused especially on London, including historic studies of urban transformation and diversity (e.g., Vaughn, 2018).

While the literature provides insights into contemporary changes in neighbourhood‐level ethnic residential segregation (e.g., Catney, 2016a; Lan et al., 2020; Catney et al., 2023), less is known about the transitions between neighbourhood states, in terms of their ethnic composition. Focusing on White neighbourhood dominance in London between 2001 and 2011, Johnston et al. (2015) showed decreases in majority White neighbourhoods, shifting towards greater interethnic mixing. An increasingly complex spatial tapestry of ethnic diversity within and beyond urban areas has been observed for multiple decades (Catney, 2016b; Catney & Lloyd, 2020; Catney et al., 2023). Recent work shows that the *process* of ethnic neighbourhood transition in England and Wales is distinctive in that the mixing of multiple groups does not promote neighbourhood succession as it does in the United States. Neighbourhoods in England and Wales that are highly diverse, such that they contain multiple ethnic groups with none in the majority, are much more likely to stay that way over successive census periods than their equivalent in the United States (Catney et al., 2021).

## 3 | MIXING IN HOUSEHOLDS, IN NEIGHBOURHOODS

Ethnic and racial mixing occurs at other scales than neighbourhoods. In the United States, theory has long held ‘marital assimilation’ among racial and ethnic groups as a key moment in the integration process. An influential report on immigrant integration described intermarriage between racially minoritised immigrants and US‐born White people as the ‘ultimate proof of integration for the former and as a sign of assimilation’ (Gordon, 1964, pp. 80–81; NAS, 2015, pp. 1–2).

This pathway, however, is more complex than it appears on the surface. While intermarriage can entail both integration in families and social networks, ‘we cannot assume that minority individuals (or couples) who have intermarried necessarily feel welcomed, or that they “belong”, in many mainstream settings’ (Song, 2009, p. 341). Navigating these issues is thus fraught, but there is no question that more people are facing these challenges today than just a few decades ago. Social attitudes towards ethnic mixing in partnering and households have relaxed, and these have translated into greater levels of mixing in all walks of life—work, school/university, leisure and residential environs—increasing the odds of the formation of mixed romantic partnerships and other relationships (Tindale & Klocker, 2018). The explosive growth of dating and roommate apps have extended the possibilities for encounter with people outside one’s ethnic group, beyond these physical realms (Rosenfeld & Thomas, 2012).

Mixing in households, in whatever form it takes, is distinctive from mixing in neighbourhoods. Households are not simply reducible to a microscale collection of people nesting within the larger public scale of the neighbourhood in which they reside. This is because of the attributes of households, their processes of formation, modification and dissolution, their mobility, and the activities that take place within them. Households^2^ form through various types of partnering and living arrangements and may grow with births, adoptions, extended family and caring arrangements, and roommates. They may also shrink and eventually dissolve as children become adults and form their own households, when partners separate or other family members or roommates leave, and through deaths. Feminist geographers note that households are a unique scale; they are intimate, private spaces where members perform gendered activities of reproduction, consumption, and domesticity (England, 2008; Marston, 2000; Wilkinson, 2014). Households are different in other ways; they—in existing or new configurations—may move to areas where household preferences are better satisfied. Life‐course events within households—childbirth, divorce, retirement—often alter these preferences and trigger moves, as well as the configurations of shared living spaces across one’s life‐course. The private space of households, their formation, composition, and daily activities beyond the home, affects life in the public space of neighbourhoods and moulds the social geography of the city (Blunt & Sheringham, 2019; Buzar et al., 2005). Our specific interest is in how ethnic mixing in the space of the home has implications for neighbourhood ethnic population mix and change.

In this analysis, we pay particular attention to the role of mixing between Whites and other ethnic groups given that White remains by far the largest ethnic category in England and Wales (and White British the largest ethnic group) (Catney et al., 2023). One way to theorise why mixed households that include a White person could be a mechanism for introducing diversity into White neighbourhoods derives from buffering theory. In its original form, buffering theory held that the presence of Asian or Latinx people in largely White neighbourhoods in the United States makes it easier for Black people to be accepted as neighbours by White residents in those spaces (Farley & Frey, 1994; Santiago, 1991). Recently, Wright and Ellis (2021) speculated that mixed‐race households could similarly ‘buffer’ White intolerance of racialised others. Such households reduce the magnitude of change posed by households where all members are not White. Some research suggests that people in racially mixed households and racially mixed people prefer racially mixed residential spaces (Caballero et al., 2008; Feng et al., 2014; Holloway et al., 2005; Smith et al., 2011; Wright et al., 2011). Other research, however, finds a gravitation towards spaces where White people predominate, especially when one of the members of the household is White (Wright et al., 2013). The presence of mixed‐ethnicity households involving one White member of the household in White residential spaces is potentially an important component of the diversification of these spaces. We expand buffering theory by considering the role of mixed‐*ethnicity* households in the diversification of White spaces.

Whiteness and its associated privileges has been subject to growing attention in the United Kingdom, particularly in relation to its complexifying and diversifying identities and hierarchies preceding and in the aftermath of Brexit (Botterill & Burrell, 2019; Clarke, 2023; Hickman & Ryan, 2020; Sime et al., 2022). Exploring the ‘Whitening’ thesis, with its origins in US studies of intermarriage, Song (2021, p. 947) questions in relation to multiracial people in Britain ‘the empirical basis of arguments that Asian/White individuals are effectively joining the White category…Whiteness and its attendant social privileges have historically gone together, and in many ways, still do. But we need to unpack what we mean by “White”, as this category now includes a significant degree of diversity’. We take up this challenge, but for neighbourhoods. Our hypothesis runs counter to the Whitening theory, arguing that White spaces have become more mixed and diverse, and rather than being attracted to White ‘spatial’ privilege, mixed households may be attracted to neighbourhoods experiencing increasing ethnic diversity—that is, to spaces losing their White homogeneity.

The diversification of majority White spaces is under‐researched in the British context. Johnston et al. (2013) found a declining proportion of people living in majority White neighbourhoods—which they defined as at least 80% White—in England and Wales between 2001 and 2011, and a corresponding growth of people living in ‘shared space[s]’ (p. 756). They point out that ‘although most of the population (still over three quarters) live in areas where Whites predominate [at 50% or more], that percentage is declining’ (Johnston et al., 2013, p. 756). They found the same trend in London, but with greater intensity: the number of White people living in neighbourhoods that were at least 80% White nearly halved in the decade. In short, the share of majority White spaces are shrinking, while shares of highly ethnically diverse neighbourhoods have grown. Catney et al. (2021) also showed that, between 1991 and 2011, neighbourhoods in England where White people form a considerable majority (‘low‐diversity White neighbourhoods’) were decreasing numerically. Most transitioned into neighbourhoods where White people still predominate, but where there is also notable ethnic diversity (‘moderate‐diversity White neighbourhoods’). In turn, moderate‐diversity White neighbourhoods constituted the most common pathway into high‐diversity neighbourhoods.

Where are households in all this? We acknowledge the growing interest in Whiteness in the United Kingdom and the relative paucity of detailed (quantitative) explorations of changing White spaces. We recognise the growth of ethnic diversity in neighbourhoods and the less well‐understood role of mixed households in neighbourhood transitions. We expand on Feng et al. (2014, p. 385), who noted that ‘most existing studies of ethnic segregation ignore the fact that the majority of adults live in couples’ and think about other household formations. Most existing studies of ethnic segregation (and diversity) do not account for the fact that the majority of adults share households with others. Research in the United States and Australia shows that mixed‐ethnicity households have meaningful effects on aggregate levels of segregation, and changes in segregation over time (Ellis et al., 2007, 2012; Tindale & Klocker, 2018). The limited consideration of the household as a unit of analysis in studies of diversity and segregation in a UK context may have masked important social and spatial processes.

## 4 | DATA AND METHODS

### 4.1 | Mixed households: Growth and types

Table 1 showcases the possibilities for exploring household mixing.^3^ Data from the Census of England and Wales provide information on the number of single‐person and multiperson households, and the proportion of the latter which are mixed ethnicity—that is, where two or more of a household’s residents belong to different ethnic groups. The ‘type’ of ethnic mixing within the household is also given, and identifies if that mixing is between household members of different generations, between partnerships, or in some other combination, examples of which might be students or lodgers.

Using Census data on household compositions for England and Wales for 2001, 2011 and 2021, Table 1a shows the potential significance of intra‐household mixing in shaping neighbourhood mixing. This level of ethnic diversity within intimate spaces is striking; and growing. While the proportion of one‐person households was stable from 2001 to 2021, multiperson households became more ethnically diverse over both decades. Households of multiple occupancy where all members were of the same ethnicity declined from roughly 64% of all households in 2001 to just under 60% in 2021, while mixed households grew from 6.4% in 2001, to nearly 9% in 2011, and just over 10% in 2021. The pace of growth of household mixing slowed a little between 2011 and 2021, and yet experienced a 25% increase (8.7%–10.1% of households). Notably, of the nearly 25 million households in England and Wales in 2021, more than one in 10 comprised people of at least two different ethnic groups. Just as increasing numbers of people claim ethnically diverse identities (Catney et al., 2023; Jivraj & Simpson, 2015), similar changes are occurring in the spaces of the home.

Table 1(b) displays the composition of mixed‐ethnicity households in 2021, and how this changed from the two decades previous. These households comprised almost 15% of all multiperson households in England and Wales in 2021, up from 9% in 2001 (bottom row of Table 1(b)). Mixed‐ethnicity partnerships have received considerably more attention than mixed households and, as discussed, this interethnic mixing is often interpreted as a barometer for improved social integration and acceptance. Yet partnership formation between two *individuals* also involves (at least) one *household*. As shown in Table 1(b), while the proportion of households that were mixed due to partnerships declined between 2001 and 2011, it grew in the subsequent decade, with over half of mixed households in 2021 comprising two people of different ethnicities living together in a partnership. These intimate unions are perhaps the most familiar form of mixing within households and reflect the considerable number of people who are in an interethnic relationship—nearly 2.3 million in England and Wales in 2011, with the share of mixed‐ethnicity couples growing from 1% to 9% between 1991 and 2011 (Berrington, 1996; ONS, 2014).

Table 1(b) sheds light on the other forms of within‐household mixing that might also shape neighbourhood change. In 2011, 25% of mixed households were made up of people who have different ethnicities between generations. An example is a two‐parent household with their offspring at home where the parents identify as White Irish, living with their White British child(ren). Other examples might include: single parents with a child of a different ethnic group; single or coupled parents with an adopted child of a different ethnic group; and natural and step parents with a child of a different ethnic group to the two parents. The number and proportion of this type of household dropped between 2011 and 2021, from over 25% to under 18%, while shares of mixing within partnerships and other forms of mixing increased. Row 2 of Table 1b (ethnic groups differing within partnerships) includes households headed by partners identifying with different ethnic groups whether or not there are different ethnic groups across generations in that household.

By 2021, interethnic mixing between partners remained the most prevalent form of household mixing. At the same time, one‐quarter of mixed‐ethnicity households involved people living together in ways other than through familial relationships, such as co‐residing students or lodgers (Table 1b, Row 3). These living arrangements are substantively different to partnerships, perhaps signifying a less significant interethnic boundary crossing than in family relationships. Yet they are another form of identifying with, and living with, difference in intimate spaces, reflecting and transforming the experience of diversity in the neighbourhoods in which they reside.

### 4.2 | Neighbourhood diversity schema

Given that two‐fifths of interethnic relationships include a White British person (ONS, 2014), our interest is in the effect mixed households have on diversity in White neighbourhoods. We identify those neighbourhoods using a schema developed by Holloway et al. (2012) for the US and adapted for the English case by Catney et al. (2021). We would ideally be able to identify which mixed households have White British members, but such data are not available. Lower layer Super Output Areas (LSOAs) are used to represent neighbourhoods. The base geography for Census data are Output Areas, which were designed to be homogenous in terms of their population and housing characteristics. OAs were aggregated into larger units—LSOAS—and are an appropriate geography for this analysis given their relative homogeneity. Their size is also advantageous over alternative geographies, avoiding excessive spatial granularity while also capturing local variation. As the boundaries of LSOAs can change each decade, this analysis is based on 2021 LSOAs. To enable comparisons using ethnic group and ethnically mixed household data from the 2001 and 2011 Censuses, it was necessary to make the counts for these earlier periods geographically consistent with the 2021 data; this process is detailed in the Supporting Information S1: Technical Appendix.

The neighbourhood schema (described in detail in Catney et al., 2021) uses an eight‐group entropy‐based measure to identify neighbourhoods that are either (i) low‐, (ii) moderate‐ or (iii) high‐diversity. The first two neighbourhood types also identify which ethnic group is largest, of White, Indian, Pakistani, Bangladeshi, Chinese, Black African, Black Caribbean and Other. An important feature of the schema for this study is that it does not centre the absence of White people in identifying diversity (as with, e.g., Johnston et al., 2013). For this eight‐group schema we do not separately identify White British and White neighbourhoods, but include in the White classification these minoritised groups: White Irish, White Gypsy or Irish Traveller, White Other and, in 2021, Roma.

We focus on two specific types of transitions: from low‐diversity White to moderately diverse White neighbourhoods; and from moderately diverse White neighbourhoods to the next most common neighbourhood type—high‐diversity—in which no ethnic group forms a majority. We do this for several reasons: (i) Many majority White neighbourhoods are changing. Low‐diversity White neighbourhoods were by far the most numerous in 2011, but their share of all neighbourhoods decreased after 1991, from around 91% in 1991, to 86% in 2001, to 79% a decade later. 2021 Census data reveal a further decline in this type of neighbourhood—to around 71% in 2021. The vast number of low‐diversity White neighbourhoods that transitioned became moderately diverse White neighbourhoods. (ii) Similarly, moderately diverse White neighbourhoods were by far the most significant source of transitions into highly diverse neighbourhoods between 1991 and 2011, and between 2011 and 2021. (iii) Once neighbourhoods transition into more diverse (yet still White‐dominated) spaces (i.e., moderate‐diversity White), they do not commonly transition back to low‐diversity White. This stability is also the case for neighbourhoods that transitioned from moderately diverse White to high‐diversity. Greater diversity is thus the direction of travel—to either high‐diversity neighbourhoods, or moderately diverse neighbourhoods dominated by a racially minoritised group, the most common of which, between 2001 and 2011 and 2011 and 2021, was to moderately‐diverse Pakistani, Indian or Bangladeshi majority neighbourhoods. We use these neighbourhood typologies and the data available on mixed households introduced in Table 1 for the rest of our analyses.

## 5 | HOUSEHOLD AND NEIGHBOURHOOD MIXING

### 5.1 | Mixed household neighbourhood types

Table 2 shows the number and percentage of each multiperson household composition for neighbourhood types in England and Wales in 2011 and 2021. To allow us to concentrate on the most common neighbourhood types, we impose a threshold of a minimum of 20 neighbourhoods for the analysis. This allows for the inclusion of 99.87% of neighbourhoods in England and Wales in 2021.^4^ The first two columns of data show the number of neighbourhoods in each neighbourhood type and the total number of households found in these neighbourhoods that are home to more than one person. The next two columns show, by neighbourhood type, the percentage of same ethnicity households, and the percentage share where the household occupants come from different ethnic groups. The final columns disaggregate mixed households by their composition and include the percentage of mixed households that are home to people who are mixed among generations, partnerships, and in other ways (such as flatmates), again by neighbourhood type. While in Table 1(b) the denominator for household compositions (‘types’ of household mixing) is the number of mixed‐ethnicity households, the denominator in Table 2 is the number of multiperson households.

Table 2 provides insight into the interplay between household and neighbourhood diversities in 2011 and 2021. The type and number of ethnically mixed households and neighbourhoods change each decade and we have to keep these dynamics in mind while reading Table 2. The fewest shares of mixed households are found in neighbourhoods with large shares of Whites (low‐diversity White). Nearly 90% of multiperson households in these neighbourhoods comprised people with the same ethnic identities in 2021. Nevertheless, there were also a substantial number of mixed households in these neighbourhoods. Indeed, over 1.2 million households were mixed ethnicity.^5^ Moreover, household mixing in low‐diversity White neighbourhoods has grown: in 2011, the proportion of multiperson households that were ethnically mixed was 8.63%; in 2021, it was 10.21.

While the share of mixed households in high‐diversity neighbourhoods decreased from 31.04 to 26.78, between 2011 and 2021, the number of mixed households in such neighbourhoods grew, from just under 200,000 in 2011 to nearly one‐quarter of a million households in 2021. The count of highly diverse neighbourhoods increased from 1515 in 2011 to 1931 in 2021. Thus, in this decade the number of mixed households increased at a faster rate than the number of high‐diversity neighbourhoods.

Moderate‐diversity white spaces have very different characteristics in relation to household mixing. In 2021, over one‐quarter of multiperson households in these neighbourhoods were ethnically mixed, and around 13% had homes with interethnic couples. Previous research demonstrated how moderate‐diversity White neighbourhoods act as important conduits into higher levels of neighbourhood diversity (Catney et al., 2021). Of the 913 neighbourhoods that became high‐diversity between 2001 and 2011, 857 were moderate‐diversity White in 2001; that is, around 94%. Between 2011 and 2021, a similar proportion of neighbourhoods transitioned from moderate‐diversity White to high‐diversity.

We cannot, in this analysis, distinguish between cause and effect— do mixed households or mixed neighbourhoods come first? We can, however, explore the extent to which household mixing might be a driver of neighbourhood change, towards greater ethnic diversity. Ellis et al. (2012, pp. 565–566) demonstrated, for the US, that ‘mixed‐race households can also drive change in segregation…if mixing at the scale of the household is increasing then segregation declines even when single‐race households prefer to live in neighbourhoods in which they are the majority’. Moderate‐diversity White neighbourhoods largely emerge due to transitions from low‐diversity White neighbourhoods, where mixed households are less common. And as many moderately diverse White neighbourhoods are pathways towards high diversity, it seems likely that, given Ellis et al. (2012), interethnic mixing at the household scale is part and parcel of the production of greater ethnic diversity at the neighbourhood scale.

Table 2 also shows that in high‐diversity neighbourhoods in 2021, nearly 27% of multiperson households were mixed. A similarly high proportion of mixed households can also be found in moderate‐diversity White and Bangladeshi neighbourhoods.

Two main points follow: First, diversity more commonly observed at the neighbourhood scale masks diversity at the household scale. This is a problem if we are to fully understand social integration, and pay due attention to the potentially trans-formative role of households in driving neighbourhood change. Could mixed households be one of the core ingredients in explaining the stability of high diversity in the English context that Catney et al. (2021) found compared to the relatively high rates of instability in such neighbourhoods found in a US context (Wright et al., 2020)?

Second, a large proportion of mixed households are found not only in highly ethnically diverse spaces, but also White majority spaces. This lends support for the hypothesis that the greater presence of mixed households yields neighbourhood diversity. The elements of neighbourhood desirability (e.g., good schools, personal safety, green space) likely do not vary much across households. Nevertheless, highly diverse neighbourhoods might be viewed as attractive for mixed unions, although it might well be that interethnic households, in particular couples, are attracted to diverse, yet still White‐dominated, neighbourhoods (Holloway et al., 2005). High‐diversity spaces, while growing in number, are, however, still relatively rare, and geographically concentrated, particularly in London (Catney et al., 2021, 2023; Johnston et al., 2015). There might thus be a tension of choice versus constraint for mixed households in their neighbourhood location preferences, who might have to reside in a moderately diverse White majority neighbourhood in the absence of more diverse neighbourhoods, particularly outside the capital.

Shifting from reading *down* the columns of Table 2 to *across* its row adds to our understanding of these geographies of intermixing. Household mixing between partnerships is the most dominant form of ethnic mixing in 2021 in five of the six neighbourhood types shown in Table 2. This balance has changed since 2011, where interethnic mixing between generations was the most common form of mixing in half of the neighbourhood types. By 2021, low‐diversity White neighbourhoods had fewer households mixed by generation, with a corresponding growth of mixing within partnerships and other forms of mixing. In high‐diversity neighbourhoods, mixing in households within interethnic partnerships accounted in 2021 for a sizable 10% of multiperson households. Perhaps most strikingly, multiperson households in moderate‐diversity White neighbourhoods had the highest proportion of mixed‐ethnicity partnerships, at over 13%.

In moderate‐diversity Bangladeshi neighbourhoods, a similarly high proportion of households (11%) were mixed due to other forms of mixing within households, such as between students or lodgers. These large shares are likely to coincide with student neighbourhoods in major towns and cities, and perhaps the particular housing landscape of places such as Tower Hamlets, where the Bangladeshi group is populous and where high levels of overcrowding occur (Elahi & Khan, 2016).

Table 2 shows that the distribution of both neighbourhood types and ethnically mixed households changed from 2011 to 2021. This makes it challenging to see decadal change in the concentration of mixed households across neighbourhood types. Table 3 restructures Table 2‘s data to show these concentrations. Unlike Table 2, the percentages in this table are shares of neighbourhood types and mixed‐ethnicity households across all neighbourhood types. For example, in 2011 78.92% of neighbourhoods were low‐diversity White and these contained 55.54% of mixed ethnicity households.

Of most interest to us is the changing distribution of mixed‐ethnicity households relative to the distribution of neighbourhood types. Before we discuss this further we first draw attention to changes in percentage share of neighbourhoods by type. Table 3 shows that two biggest changes were the decline in low‐diversity White, and the rise of moderate‐diversity White, neighbourhoods. The most substantial change after these is the increased share of neighbourhoods classified as high‐diversity. Low‐diversity White neighbourhoods were home to just over half of all mixed‐ethnicity households in 2021. In the same year, well over a third of all mixed‐ethnicity households lived in moderate‐diversity White residential areas. Just under 10% of these households lived in high‐diversity neighbourhoods. That most mixed‐ethnicity households reside in White neighbourhoods is unsurprising given that these spaces, low‐ and moderate‐diversity, account for over 90% of all neighbourhoods.

The relative concentration of mixed‐ethnicity households across neighbourhood types reveals a different story than a focus on their shares alone. Here we compare the share of mixed‐ethnicity households in a neighbourhood type to the share of neighbourhood types (see last column of Table 3). If mixed‐ethnicity households are distributed across neighbourhood types in proportion to the distribution of those types then this ratio will equal one. Ratio values greater than one indicate a concentration of mixed‐ethnicity households in a given type of neighbourhood. For example, a ratio of two means the share of mixed‐ethnicity households in that neighbourhood type is twice the share that type has of all neighbourhoods. Ratio values less than one mean the opposite: a lack of concentration of mixed‐ethnicity households. This method accounts for changes in the share of neighbourhood types over time. It can show whether the relative concentration of mixed ethnicity households across neighbourhood types is stable.

Using this ratio we see that mixed‐ethnicity households have the highest levels of concentration in moderate‐diversity White and highly‐diverse neighbourhoods. Even though low‐diversity White neighbourhoods have the highest share of mixed households, these are also the places with the most marked lack of concentration of this household type. In fact, these neighbourhoods are the only type with a consistent lack of concentration in both years, with barely any change in relative concentration over the decade. For neighbourhood types with ratios greater than one in 2011, the decadal trend is a decline in concentration. This is most pronounced in moderate‐diversity White and highly diverse neighbourhoods. These two neighbourhood types are the spaces in which mixed households are most disproportionately concentrated in both 2011 and 2021, but the level of concentration in both dipped below two and converged over this decade. Only one neighbourhood type transitioned from a modest concentration to lack of concentration over the decade: moderate‐diversity Pakistani. The overall impression is that mixed‐ethnicity households remain disproportionately concentrated in almost all neighbourhood types other than low‐diversity White. That level of concentration has declined since 2011, however, as these other than low‐diversity White residential spaces increased their share of all neighbourhoods.

**FIGURE 1:**
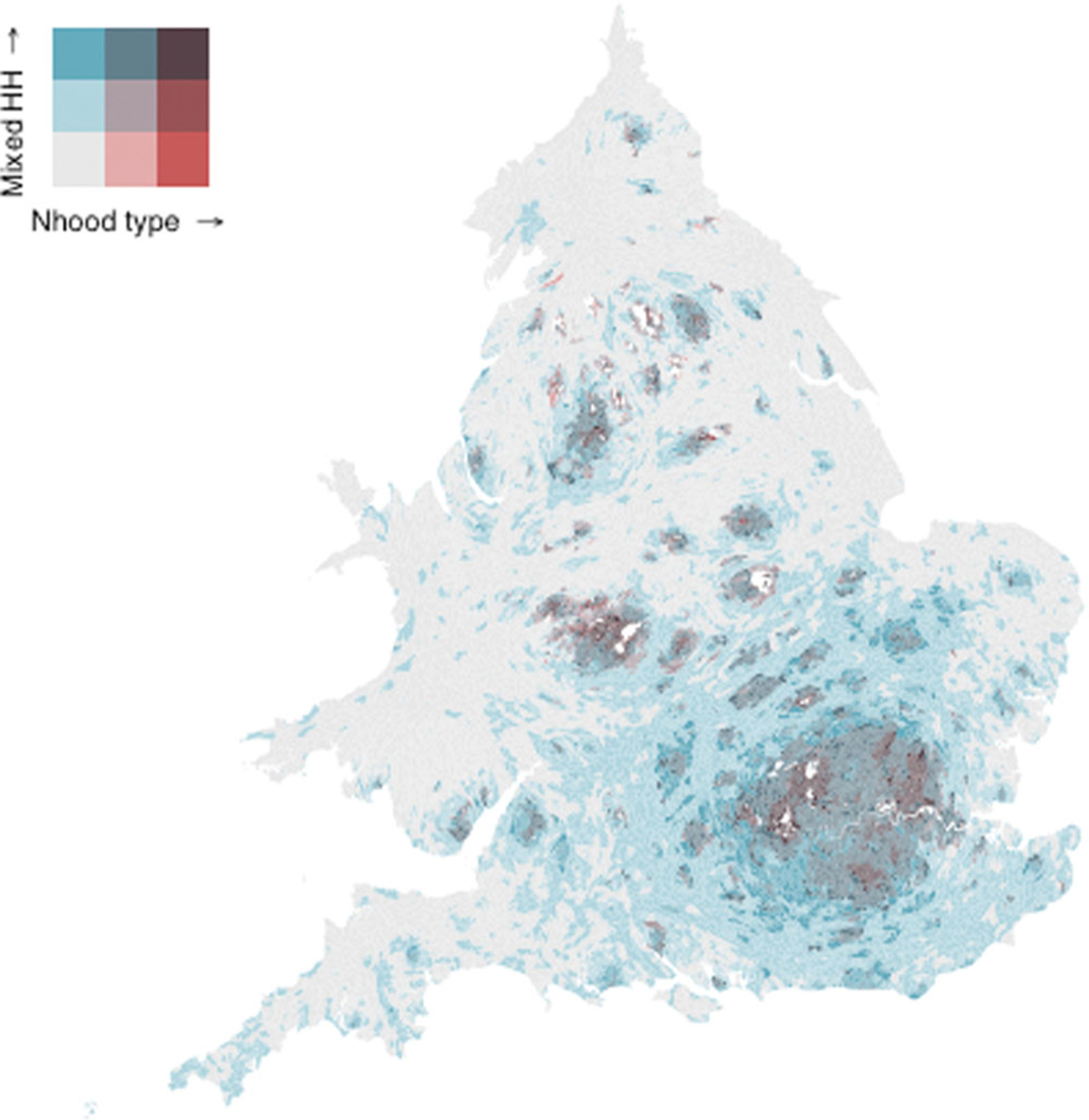
Bivariate map of proportion of mixed‐ethnicity households in low‐diversity White, moderate‐Diversity White, and high‐diversity neighbourhoods, 2021, England and Wales. Note that this map is a cartogram, with LSOAs scaled by the square root of their original geographical area (see Catney et al., 2023). HHs, households; N’hood, neighbourhood. Source: 2021 Census, Tables TS021 and TS023. Authors’ own calculations.

Figure 1 offers an alternative take on these connections between neighbourhood and household. This bivariate map of LSOAs in England and Wales shows, concurrently, the spatial distributions of mixed households and White‐dominated and high‐diversity residential neighbourhoods in 2021. Information about two variables is shown for each neighbourhood: their diversity type (*x*‐axis), and their percentage of mixed households as a proportion of the number of multiperson households (*y*‐axis). There are three categories for each variable: neighbourhoods are low‐diversity White, moderate‐diversity White, or high‐diversity, and have high, medium, or low proportions of mixed‐ethnicity households.^6^ Our focus on both White and highly ethnically diverse spaces means that other types of neighbourhoods (e.g., moderate‐diversity Pakistani, low‐diversity Indian) are not represented, and are coloured white on the map. Even with those subtractions, Figure 1 includes 98.19% of all neighbourhoods. To help read the map, consider two examples: neighbourhoods shaded the darkest colour (top right cell) are high‐diversity neighbourhoods with the highest proportion of mixed households. Neighbourhoods shaded the lightest blue (bottom left cell) are low‐diversity White neighbourhoods with the lowest proportion of mixed households.

Low‐diversity white spaces were the dominant geography across much of rural England and Wales. Moderately‐diverse White neighbourhoods can be found, typically, within and nearby urban places. With this context in mind, Figure 1 shows that high‐diversity spaces in 2021 were urban, located in major centres like London, Birmingham and Manchester. The geography of mixed households does not map neatly onto that of the neighbourhood types. We might have expected consistently higher levels of interethnic household mixing in urban rather than suburban and rural areas. Yet we find a much more complicated spatial pattern, with high and moderate levels of mixed household formations in low‐diversity White neighbourhoods found principally in the south and south‐east of England, as well as several coastal areas. While large swathes of England and Wales are low‐diversity White with low levels of mixed households, the map is suggestive of new spaces of layered mixing of people within households, within neighbourhoods. We see many examples of moderate and even proportionately high levels of mixed households within all neighbourhoods types. These intricate geographies of ethnic mixing reflect, in part, residential preferences for diverse spaces for people in mixed households, but their complexity implies that this is not straightforward. People in (mixed) households likely seek neighbourhoods that might not necessarily be diverse, but offer desirable attributes and amenities (good schools, green spaces, etc.). Added to this, residential preferences are of course constrained by availability, for example, of diverse spaces where these are desired, and of where housing is affordable.

### 5.2 | Mixed households and neighbourhood transitions, 2001–2011–2021

We next explore decadal neighbourhood transitions, 2001–2011–2021. We hypothesise that neighbourhoods that were high‐diversity, or transitioned to that state, have a higher proportion of mixed households (at the beginning of each decade) than other neighbourhood types. Table 4(a) shows the percentage of mixed households (as a proportion of all multiperson households in 2001) in each neighbourhood transition type that met a threshold of 1000 households in 2001.^7^ Table 4(b) shows the same, for transitions from 2011 to 2021 (using 2011 as the base). To obtain this measure, the number of mixed households in a given neighbourhood transition type are divided by the total number of households in that transition type, which are then expressed as a percentage. As an example, 25% of households in neighbourhoods that stayed as moderate‐diversity White between 2001 and 2011 were mixed ethnicity. The threshold of 1000 households allows us to ignore neighbourhood transition types where the number of households is small, and where some of the transition types might represent just one neighbourhood. The neighbourhood transition types that meet this threshold for each year are shown in Table 4(a) and (b); blank cells did not meet the threshold for that particular neighbourhood transition type.

First and foremost, Table 4 shows that mixed households may play a role in the maintenance of moderate diversity for White and Asian majority neighbourhoods. The diversity of these spaces—and the *persistence* of this diversity—appears to be partly a function of the diversity within *households* in these neighbourhoods, and not just *between* mono‐ethnic households. Second, reading across rows in Table 4, neighbourhoods that transitioned into high‐diversity typically had higher proportions of mixed households than for other transition types for neighbourhoods. Being home to a high proportion of mixed households appears to push a neighbourhood to transition into high diversity, and act to maintain it. Third, and relatedly, moderate‐diversity White neighbourhoods are something of a special case. Neighbourhoods that remained moderately diverse White between 2001 and 2011, and between 2011 and 2021, had a large share of mixed households; indeed, at least one‐quarter of these neighbourhoods had homes comprising people of different ethnicities. A similar proportion of mixed households can be found in neighbourhoods that tipped from high‐diversity to moderate‐diversity White.

This table provides some insight into the increasing ethnic complexity of White majority spaces. Not only are these neighbourhoods home to a considerable proportion of people who are not White, living in different households in the same street, but they are also spaces where there is considerable mixing *within* households. Data constraints prevent us from determining what the composition of that within household mixing might look like,^8^ but given the high proportion of mixing between White Britons and people in other ethnic groups (ONS, 2014), it is reasonable to claim that the White British population in moderately‐diverse White neighbourhoods are very much a part of these layers of interethnic mixing. The same can be said for moderately diverse White neighbourhoods that transition into high‐diversity between 2001 and 2011, and between 2011 and 2021; these neighbourhoods were home to a similar proportion of mixed households. Moderately diverse White neighbourhoods are the most common source for transitions into high‐diversity; this might suggest that within‐household mixing helps shape transitions into greater neighbourhood diversity from these spaces.

## 6 | CONCLUSIONS

Our paper, written as part of the *Geographies of Ethnic Diversity and Inequalities (GEDI)*^9^ project, explored some interconnections between ethnic mixing at the scales of households and neighbourhoods in England and Wales between 2001, 2011 and 2021. Mixed‐ethnicity households rose from around 6.5% of all households in 2001, to just under 9% in 2011, to over 10% in 2021; and increased ethnic mixing characterised neighbourhoods that transitioned in England and Wales in this period (Catney et al., 2021). We examined the interplay between these household and neighbourhood trends, with a particular focus on household mixing in White‐dominated neighbourhoods. We chose this emphasis because such neighbourhoods comprise a large majority of residential spaces in England and Wales, and are the biggest source of transitions to highly diverse neighbourhoods where no one group dominates (Catney et al., 2021). Our analysis asked whether the presence of mixed households in White (and other group) neighbourhoods is associated with the diversification of those spaces.

We found that the share of mixed‐ethnicity households increases with the level of diversity in neighbourhoods. Just over a quarter of multiple occupancy households in moderate‐diversity White neighbourhoods were mixed, compared to 10% in low‐diversity White neighbourhoods. In high‐diversity neighbourhoods, around 30% of multiperson households were ethnically mixed, growing to nearly a quarter of a million households by 2021. Increased diversity in neighbourhood space is bound‐up with greater rates of mixing in households, suggesting a connection between the diversification of the public space of the street and the private space of the home. That household mixing rates in moderate‐diversity White neighbourhoods are 2.5 times higher than in low‐diversity White neighbourhoods demonstrates that interethnic household presence is a feature of diversified White‐dominated residential space.

The data we used prevents us from clearly discerning cause and effect between household mixing and neighbourhood transition. Nevertheless, we can identify associations between rates of household mixing in 2001 and transitions 10 years hence, and likewise between 2011 and 2021. Neighbourhoods that transitioned into high‐diversity between 2001 and 2011 generally had a higher percentage of mixed households in 2001 than those that remained the same or transitioned into some other (less diverse) state. The same pattern is observable for transitions between 2011 and 2021. Moderate‐diversity White neighbourhoods are an interesting exception here. Those that remained moderately diverse White between 2001 and 2011 had a higher percentage of mixed households than those that transitioned to another state, including high‐diversity. For 2011–2021, neighbourhoods that stayed moderate‐diversity White or transitioned from that neighbourhood type to high‐diversity shared the same levels of mixed households (with proportions higher than for 2001–2011, at over one‐quarter of multi‐person households). On the whole, household mixing solidifies the stability of white‐dominated residential spaces that have already diversified.

Our findings also revealed some interesting shifts in the composition of ethnic mixing within home spaces, where mixing between partnerships has increased, in contrast to mixing between generations, which declined. Alongside these changes, the number of households that are mixed through other arrangements, such as flat‐ sharing between friends, has grown over time. These findings build a platform for future studies of the nature of mixed‐ethnicity families (Campion & Lewis, 2022) and friends, and are potentially important signifiers of mixed‐ethnicity futures.

Just as White ethnic identities have diversified over time (Catney et al., 2023; Hickman & Ryan, 2020), the composition of what we here classified as White neighbourhoods will also have become increasingly complex, home to growing numbers of people of White minoritised ethnic groups. Unpacking this diversification would deepen our understanding of the characteristics of the transitions taking place in (White) residential spaces. Subsequent research should also seek out data that provide more detail on household composition, especially ethnic composition, generation, socioeconomic status, and stage in the life course.

A large proportion of interethnic relationships involve a White British person (ONS, 2014). Knowing where these and other mixed ethnic households live, defined by combinations of ethnic groups,^10^ would show whether specific types of household mixing are associated with types of neighbourhood transition. Mixed partnership households may contain children or not and these children could have the same or a different ethnic group to one or both of the parents. The reason for the mixedness in the household is important to explore further in terms of how much of a boundary crossing in terms of culture it is creating and, therefore, the impact it might have on perpetuating diversity (or not) (Voas, 2009) within the neighbourhood. For example, if most of the intergenerational households are a result of children belonging to the ethnic majority, this will reduce residential diversity in most places in England and Wales. Socio-economic status and life‐cycle status will likely condition these effects. Appeals in the Population Geography literature to better integrate household processes into considerations of household structure and neighbourhood change (Buzar et al., 2005; Wright et al., 2003) have gone largely unanswered. Our findings add volume to these calls for more research into these dynamics.

## Supplementary Material

Supporting information

## Figures and Tables

**TABLE 1:** (a) Household types and (b) mixed‐ethnicity household combinations, 2001, 2011 and 2021, England and Wales.

(a)						
Household type	*n* 2001	% 2001	*n* 2011	% 2011	*n* 2021	% 2021
One‐person	6,502,612	30.02	7,067,261	30.25	7,481,788	30.19
Multiperson, same ethnicity	13,780,138	63.62	14,274,034	61.09	14,791,805	59.68
Multiperson, mixed‐ethnicity	1,377,725	6.36	2,024,749	8.67	2,509,604	10.13
*Total households*	*21,660,475*	*100*	*23,366,044*	*100*	*24,783,197*	*100*
(b)						
Mixed‐ethnicity household composition	*n* 2001	% 2001	*n* 2011	% 2011	*n* 2021	% 2021
Ethnic groups differ between generations but not within any partnerships	328,501	23.84	513,116	25.34	435,883	17.37
Ethnic groups differ within partnerships	788,841	57.26	1,052,420	51.98	1,424,981	56.78
Any other combination of multiple ethnic identities	260,383	18.90	459,213	22.68	648,740	25.85
*Total mixed‐ethnicity households*	1,377,725	100 *(9% of multiperson HHs)*	*2,024,749*	*100 (12% of multiperson HHs)*	*2,509,604*	*100 (15% of multiperson HHs)*

*Note*: Different ethnic groups within partnerships are reported as such whether or not there are different ethnic groups between generations in the household. Due to rounding, totals might not sum to exactly 100%.

Abbreviation: HHs, households.

*Source*: 2001 Census, Table UV069; 2011 Census, Table QS202; 2021 Census, Table TS023. Authors’ own calculations.

**TABLE 2:** Number and percentage of multiperson household compositions by neighbourhood type, 2011 and 2021, England and Wales.

Neighbourhood type	Year	N’hoods *(total* = *35,672)*	Total multiperson HHs	% Same ethnicity HHs	% Total mixed‐ethnicity HHs	% Mixed HH generations	% Mixed HH partnerships	% Mixed HH other
Low‐diversity White	2011	28,154	13,030,824	91.37	8.63	1.84	5.28	1.51
	2021	25,488	12,442,769	89.79	10.21	1.53	6.65	2.03
Moderate‐diversity White	2011	5537	2,436,859	72.66	27.34	7.47	11.95	7.93
	2021	7608	3,658,711	74.05	25.95	4.80	13.27	7.87
Moderate‐diversity Indian	2011	159	66,528	82.13	17.87	6.61	5.95	5.31
	2021	204	94,196	84.38	15.62	2.84	6.62	6.16
Moderate‐diversity Pakistani	2011	228	92,263	83.22	16.78	7.51	5.13	4.14
	2021	326	142,930	86.20	13.80	3.12	5.66	5.02
Moderate‐diversity Bangladeshi	2011	50	19,552	76.83	23.17	6.01	7.37	9.79
	2021	68	30,915	76.67	23.33	3.72	8.48	11.13
High‐diversity	2011	1515	640,775	68.96	31.04	12.09	9.76	9.20
	2021	1931	909,738	73.22	26.78	6.61	10.30	9.87

*Note*: Different ethnic groups within partnerships are reported as such whether or not there are different ethnic groups between generations in the household. Due to rounding, totals might not sum to exactly 100%. N’hoods refers to neighbourhoods. HHs refers to households. A threshold of a minimum of 20 neighbourhoods is imposed on the analysis. This omits the following neighbourhood types: moderate‐diversity Black African (2011 *n* = 3; 2021 *n* = 12), low‐diversity Indian (9; 14), low‐diversity Pakistani (6; 6), low‐diversity Bangladeshi (2; 1) moderate‐diversity Other (0; 1).

*Source*: 2011 Census, Tables KS201 and QS202; 2021 Census, Tables TS021 and TS023. Authors’ own calculations.

**TABLE 3:** Percentage shares of mixed‐ethnicity households by neighbourhood type, 2011 and 2021, England and Wales.

Neighbourhood type	Year	N’hoods (total = 35,672)	% Share of n’hoods	% Share mixed‐ethnicity HHs	% Share mixed‐ethnicity HHs/% Share of n’hoods
Low‐diversity White	2011	28,154	78.92	55.54	0.70
	2021	25,488	71.45	50.62	0.71
Moderate‐diversity White	2011	5537	15.52	32.90	2.12
	2021	7608	21.33	37.83	1.77
Moderate‐diversity Indian	2011	159	0.45	0.59	1.32
	2021	204	0.57	0.59	1.03
Moderate‐diversity Pakistani	2011	228	0.64	0.76	1.20
	2021	326	0.91	0.79	0.86
Moderate‐diversity Bangladeshi	2011	50	0.14	0.22	1.60
	2021	68	0.19	0.29	1.51
High‐diversity	2011	1515	4.25	9.82	2.31
	2021	1931	5.41	9.71	1.79

*Note*: N’hoods refers to neighbourhoods. HHs refers to households.

*Source*: 2011 Census, Tables KS201 and QS202; 2021 Census, Tables TS021 and TS023. Authors’ own calculations.

**TABLE 4:** Percentage of mixed‐ethnicity households by neighbourhood transition type, (a) 2001–2011 and (b) 2011–2021, England and Wales.

(a)							
2011							
2001	Neighbourhood type	Low‐diversity White	Moderate‐diversity White	Moderate‐diversity Indian	Moderate‐diversity Pakistani	Moderate‐diversity Bangladeshi	High‐diversity
	Low‐diversity White	6.41	15.54		11.92		
	Moderate‐diversity White	22.44	25.01	15.27	14.39	20.64	23.88
	Moderate‐diversity Indian			15.70			19.09
	Moderate‐diversity Pakistani				17.45		19.99
	Moderate‐diversity Bangladeshi		19.73			19.47	
	High‐diversity		29.66	20.71	22.28		25.18
(b)							
2021							
2011	Neighbourhood type	Low‐diversity White	Moderate‐diversity White	Moderate‐diversity Indian	Moderate‐diversity Pakistani	Moderate‐diversity Bangladeshi	High‐diversity
	Low‐diversity White	7.81	16.86				
	Moderate‐diversity White	21.75	27.53	17.19	13.89	30.65	27.95
	Moderate‐diversity Indian			17.93	11.93		20.01
	Moderate‐diversity Pakistani				16.72		18.65
	Moderate‐diversity Bangladeshi					23.14	
	High‐diversity		36.23	23.82	23.60	26.82	31.13

*Note*: blank cells did not meet the threshold of 1000 households for that particular neighbourhood transition type. The percentages in each table refer to the earlier year for the transitions (i.e., 2001 households for Table 4a and 2011 households for Table 4b).

*Source*: 2001 Census, Tables KS006 and UV069; 2011 Census, Tables KS201 and QS202; 2021 Census, Tables TS021 and TS023. Authors’ own calculations.

## Data Availability

The data that support the findings of this study were derived from the following resources available in the public domain:—2001 Census, https://casweb.ukdataservice.ac.uk/—2011 Census, https://www.nomisweb.co.uk/—2021 Census, https://www.nomisweb.co.uk/—2021 boundary data, https://www.ons.gov.uk/methodology/geography/geographicalproducts/digitalboundaries

## Footnotes

1 Authors’ own calculation of 2021 Census Table TS023 (Crown Copyright).

2 Households, the basic sociodemographic unit of society, are defined as an individual living alone (Wilkinson, 2014) or a group of people sharing an enclosed living space.

3 All analysis and data processing were undertaken using the R computing environment (R Core Team, 2022). The map in Figure 1 was produced using the R package biscale (Prener et al., 2022).

4 99.92% of neighbourhoods are included in 2011. The omitted neighbourhood types are listed as a note in Table 2.

5 12,442,769 × 0.1021 = 1,270,407. ‘White’ in our schema includes White British as well as minority White identities—Irish, Gypsy/Traveller, Other, and, in 2021, Roma—and it might be expected that much of this interethnic household mixing involves at least one White Briton and a person who identifies as, for example, White Irish or Other White, given the high rates of interethnic partnerships between people in these ethnic groups (ONS, 2014).

6 Split into three categories with approximately the same number of neighbourhoods; that is, one‐third of neighbourhoods are in each class.

7 In addition to the threshold of 20 neighbourhoods, discussed in relation to Table 2.

8 An additional 2021 Census table on the composition of mixed ethnicity households centres on which households are not mixed, by broad ethnic category: all household members are White, Asian, Black, Mixed, or Other only. The table provides counts of households which are home to two, or three or more, ethnic groups (without stating which). These data are available for 2021 only (not 2001 or 2011). While related to the theme of this paper, these data tell us more about which groups mix *less* than about which groups mix *more*. A further data publication (not available at the time of this article’s publication) will provide more detailed information on the combination of household mixing within (e.g., two or more Asian groups in household) and between (e.g., White and Mixed; Black and Other—only for households home to two ethnic groups) these broad ethnic categories, but not by type of mixing (within partnerships, between generations, etc.). This information will offer interesting future possibilities to explore the composition of household mixing.

9 Funded by the Economic and Social Research Council (award ES/W012499/1): www.gedi.ac.uk

10 See footnote 8.
