# Supplementary material for: Changes in mixed ethnicity households and neighbourhood transitions in England and Wales
Source: Popul Space Place. Author manuscript; Available in PMC 2024 Aug 8. (PMC11309028; doi:10.1002/psp.2745)
Supplement: Supporting information [file NIHMS2009200-supplement-Supporting_information.docx]

**Supporting Information S1: Technical Appendix**

**Changes in Mixed Ethnicity Households and Neighbourhood Transitions**

**in England and Wales**

**Gemma Catney | Mark Ellis | Richard Wright**

Transferring population and housing counts from 2001 LSOAs to 2021 LSOAs was achieved using areal weighting by overlaying the 2001 LSOAs and 2021 LSOAs and then overlaying postcode locations for 2001 onto the overlapping LSOA areas. The postcode densities were then used to determine the proportion of people or households transferred from each 2001 LSOA to each overlapping 2021 LSOA segment. The transferred counts were then aggregated across 2021 LSOAs. For 2011 LSOAs, the process was different: the 2011–21 ONS lookup table was used to identify LSOAs which were unchanged between 2011 and 2021. These unchanged LSOAs were removed and areal weighing was applied to all LSOAs which had changed between 2011 and 2021. These estimates and the values for the unchanged zones were then merged (see also Catney et al., 2023).
